# A General Business Model for Marine Reserves

**DOI:** 10.1371/journal.pone.0058799

**Published:** 2013-04-03

**Authors:** Enric Sala, Christopher Costello, Dawn Dougherty, Geoffrey Heal, Kieran Kelleher, Jason H. Murray, Andrew A. Rosenberg, Rashid Sumaila

**Affiliations:** 1 National Geographic Society, Washington, D. C., United States of America; 2 Centre d'Estudis Avançats de Blanes, Consejo Superior de Investigaciones Científicas, Blanes, Spain; 3 Bren School of Environmental Science & Management, University of California Santa Barbara, Santa Barbara, California, United States of America; 4 Visiting Professor, Laboratoire Montpeillerain d'Economie Théorique et Appliquée, Montpellier, France; 5 Columbia Business School, Columbia University, New York, New York, United States of America; 6 The World Bank, Washington, D. C., United States of America; 7 School of the Environment, University of South Carolina, Columbia, South Carolina, United States of America; 8 Union of Concerned Scientists, Cambridge, Massachusetts, United States of America; 9 Fisheries Centre, University of British Columbia, Vancouver, British Columbia, Canada; Aristotle University of Thessaloniki, Greece

## Abstract

Marine reserves are an effective tool for protecting biodiversity locally, with potential economic benefits including enhancement of local fisheries, increased tourism, and maintenance of ecosystem services. However, fishing communities often fear short-term income losses associated with closures, and thus may oppose marine reserves. Here we review empirical data and develop bioeconomic models to show that the value of marine reserves (enhanced adjacent fishing + tourism) may often exceed the pre-reserve value, and that economic benefits can offset the costs in as little as five years. These results suggest the need for a new business model for creating and managing reserves, which could pay for themselves and turn a profit for stakeholder groups. Our model could be expanded to include ecosystem services and other benefits, and it provides a general framework to estimate costs and benefits of reserves and to develop such business models.

## Introduction

Marine protected areas (MPAs) are intertidal and/or subtidal areas that have been reserved by law or other effective means to protect part or all of the enclosed environment, including water, flora, fauna, and historical and cultural features [1]. MPAs were initially proposed as a means to preserve marine biodiversity and unique habitats, and as an opportunity for recreation, education and research. Nevertheless, in the last two decades much of the literature has focused on whether MPAs enhance nearby fisheries and produce economic returns [2], [3]. There are many types of MPAs, from areas where most fishing is allowed to no-take marine reserves where fishing is prohibited. Because it is difficult to compare the benefits of areas with different levels of protection, here we focus on no-take marine reserves only (“marine reserves” hereafter). The literature is now quite clear about the conditions under which marine reserves produce economic and/or ecological benefits (e.g., [4], [5]). Yet in focusing almost exclusively on fisheries, this literature has ignored other, perhaps more important, aspects of the value of marine reserves. A prime example is the tourism value of marine reserves which may increase over time as biomass and diversity increase within the borders of a marine reserve. Simultaneously accounting for these, and other economic effects, allows us to create a general model that provides the foundation for a business case for marine reserves, taking care to estimate the dynamics of payoffs from reserve implementation. Globally, assembling economic arguments for, or against, marine reserves will be crucial for determining if, and how, to achieve the targets of the Convention of Biological Diversity that call for protection of 10% of the ocean (http://www.cbd.int/sp).

Here we synthesize information on the ecological and economic benefits of marine reserves, and use bio-economic modeling to show how marine reserves can be created and managed in a financially self-sustaining manner. This model incorporates both fishery and tourism benefits over time following the designation of a marine reserve.

### Ecological benefits of marine reserves

A review of peer-reviewed studies on 124 marine reserves in 29 countries showed that, on average, marine reserves cause increases of 21% in the number of species, 28% in the size of organisms, 166% in density (number of individuals per unit area), and a remarkable 446% in biomass, relative to unprotected areas nearby [4]. However, the increase in biomass of predatory fish can be greater than the above averages [6], [7], [8]. The increase in the biomass of predators has been shown to produce a re-accommodation of the food web, shifting from a degraded state typical of intensely fished sites to a more complex, mature state. These food web changes can enhance ecosystem resilience by promoting the recovery of populations of functionally important species (i.e. strong interactors [9]).

Fisheries may benefit from reserves when they help replenish nearby habitats through spillover of adult organisms and dispersal of larvae. The increase in the biomass of commercial species inside marine reserves has been shown to increase reproductive output (e.g., [10], [11]), as long as the reproductive grounds are included in the reserves. A review by Lester et al. [4] showed that areas outside reserves showed a significant increase in biomass after the reserve was in place, possibly through the spillover of adults and/or the export of larvae. Empirical studies also show that higher abundances inside reserves can lead to spillover of adults to nearby fished areas (Table 1). Spillover at small scales is common for commercial species that respond positively to reserve protection [12]. Empirical evidence on the ability of reserves to replenish fished areas through larval dispersal is limited, partly because of methodological/sampling issues [13], but there are some remarkable examples (Table 1).

**Table 1:** Examples of economic benefits of marine reserves from fishing enhancement and tourism.

Fishing			
*Area*	*Benefits*	*Observations*	*References*
Apo Marine Reserve, Philippines	Enhancement of catch of jacks and surgeonfish	Less fishing effort brought higher catch rates	[19]
Columbretes Islands Marine Reserve, Spain	Net gain of >10% in weight of the local lobster fishery catch	Caused by annual lobster spillover of 7% of the protected population. Benefits outweighed the costs of the reserve creation	[50]
Soufrière Marine Management Area, Saint Lucia	Increased adjacent catches by 46–90%	In only 5 years, despite a 35% decrease in area of fishing grounds	[21]
Sinai Peninsula Marine Reserves, Egypt	66% increase in catch per unit effort	Within only five years of the creation of the reserves	[51]
Mombasa Marine National Park	Fisher income near reserve 135% higher than in open access areas	Profits increased despite heavy fishing, diverse gear and catch, poverty, and unregulated markets	[24]
Ucunivanua marine reserve, Fiji	Clams became 7 more abundant in the adjacent fished area	After only 5 years of protection. Caused by larval dispersal.	[13], [40]
Georges Bank fishery closure	Scallop recruitment increased around the closed area	Scallop biomass increased over 14 times over 4 years in the closed area, and produced significant larval dispersal	[52]

Tourism			
*Area*	*Annual revenue*	*Observations*	*References*
Cabo Pulmo National Park, Mexico	$12,000 per capita	Higher than in most coastal communities in Mexico	[53]
Saba Marine Park, Netherland Antilles	$3 million	22% of the local economy	[54]
Mombasa Marine National Park, Kenya	$3.5 million km^−2^	350 times higher than fishing revenue	[55] [24]
Medes Islands Marine Reserve, Spain	€10 million	In only 94 ha of no-take area. 20 times higher than fishing revenue	[31]
Great Barrier Reef Marine Park, Australia	AU$5.5 billion	36 times greater than income from commercial fishing, plus 54,000 full time jobs	[37]

### Economic benefits of marine reserves

Marine reserves can provide economic benefits through tourism (diving, snorkeling, glass bottom boats), fishing (increase or stabilization of catch around reserves), and other services, some of which are difficult to quantify (e.g., insurance value, local amenity value, storm protection, political value, intangible capital). A primary concern among fishermen is the loss of fishing grounds and yields that may occur when marine reserves are implemented; these effects may not be offset by the increase in spillover and dispersal of larvae provided by the reserves [14]. An additional concern is that establishing reserves may disadvantage some fishermen such as local smaller vessels with less potential to work farther afield, to benefit fishers in other areas or with greater mobility [15].

#### Tourism

The increase in marine life inside marine reserves, in particular large fish, is the main attraction for divers and other tourists, which can bring revenue disproportionately higher than fishing (Table 1). In the wider Caribbean and Pacific coast of Central America, for instance, 50% of all dives (7.5 million dives annually) take place within marine protected areas [16], even though only 4% of Caribbean coral reefs are in MPAs rated as “good” or “partially effective” [17]. This strongly indicates the interest of divers to frequent areas with more abundant marine life. Although no data exist on the general relationship between fish biomass and diver frequentation, there is a clear preference for diving in MPAs because of the expectation of encountering more abundant marine life within their boundaries.

#### Fishing

Well-enforced marine reserves can increase adjacent fishery catches (Table 1). At small scales (on average within 1 km from the reserve boundary), local fisheries would not be sustainable without the reserves in 12 of 14 cases studied, and spillover offsets losses in catch due to the creation of the reserve in the other two cases [12]. For a full review of the effects of marine reserves on local fisheries see [18]. In addition to enhancing or ensuring sustainable yield, marine reserves can also increase the long-term profitability of fisheries (Table 1). It is important to note that the data are consistent with perceptions of the status of the fishery by the local community. In the Apo Marine Reserve in the Philippines, for example, 67–100% of the fishers interviewed believed that the fishery was improved by the presence of the reserve [19].

An additional value for fishing of marine reserves concerns catch-and-release recreational fishing inside the reserves, which may be compatible with the reserves provided that ecological impacts can be minimized [20]. Although recreational catch-and-release angling causes some fish mortality, it is considered an amenity value, and it can bring more revenue than commercial fishing to local communities. A good example is the well-regulated fly-fishing operation at the Jardines de la Reina Marine Natural Park, Cuba, which has an annual quota of sport fishers and provides a significant revenue stream. Alongside diving, fishing revenues help cover the management costs of the reserve and provide employment for Cuban fishing guides.

Recreational fishing outside reserves may also benefits from spillover. In Florida, the no-take areas in the Merritt Island National Wildlife Refuge have supplied increasing numbers of world record–sized fish to adjacent recreational fisheries since the 1970s [21].

#### Other services

Marine reserves help preserve and restore biodiversity at many levels (e.g., how many species and how many individuals of each species, and structure of the biogenic habitat; [22]). A meta-analysis showed that the increase of species diversity in marine reserves was associated with large increases in fisheries productivity, a reduction in the variability of aggregate fish biomass (which helps reduce uncertainty in fisheries), and an increase in resistance and recovery after natural disturbances from storms and thermal stress [5]. By restoring biodiversity, reserves enhance the productivity and reliability of the good and services that the ocean provides for humanity.

One of the major reasons marine reserves are not more common is that marine ecosystems are typically dominated by single uses such as fishing [23]. Yet the amenity value of marine resources protected in marine reserves (via tourism) is often greater than the commodity value of these resources (via fishing), as the examples above show. In addition, there are other non-commodified goods and services provided by marine ecosystems that can be enhanced by marine reserves. Generally, there is a lack of the non-market data required to quantify the value of these goods and services and therefore these benefits are often taken for granted [24].

By protecting coastal ecosystems such as mangroves, marshes and seagrass beds that are threatened by coastal development, aquaculture, agriculture and wood production, marine reserves can play a significant role in protecting some of the most efficient natural carbon sinks on the planet [25], enhancing coastal protection from storms [26], and ensuring the supply of fish to nearby fisheries [27]. For example, the value of one hectare of mangrove per year is up to $37,500 as a nursery for commercial fishes that will later recruit into adjacent fisheries [27], $18,000 as gross carbon credit revenue potential (assuming a carbon price of $15/t CO_2_e) [28], and $10,821 as storm protection service [29], in addition to the protection of human life on coastal areas prone to tropical storms. In contrast, the net economic return of one hectare of mangrove converted into a shrimp farm in Thailand was only up to $1220 per year in 1997–2004 [29].

Despite the increasing amount of evidence of the benefits provided by marine reserves, there are issues that have impeded the creation of marine reserves as a tool that yields economic profitability. The major economic arguments against marine reserve creation are short-term loss of fishing catch and revenue because of the closure of a fraction of the fishing grounds, and displacement of fishing effort to unprotected areas. The latter has not been a significant issue to date on a global scale because only less than 1% of the ocean is protected in marine reserves. Marine reserves are also criticized as insufficient tools for managing fisheries. It is important to note that the current research does not suggest the replacement of alternative fishery management tools. Marine reserves provide the myriad benefits described above and may further complement traditional fishery management measures in the long run. The next section presents a simulation model of the time path of marine reserve benefits and costs.

## Methods

Creating marine reserves can be an economically optimal solution when the combined value derived from tourism, the enhancement of local fisheries (via spillover from the reserve) and other services (see above) outweighs the value of any single use in isolation in the now-protected area. In what follows we develop a general dynamic model to simultaneously analyze these quantifiable effects of marine reserves on economic welfare. In particular, we develop a bio-economic model to simulate the value of a fishery and the value of tourism over time for a fishery that implements a no-take zone. We then develop a focused case study to illustrate the economic effects of marine reserves.

### Biological model

#### Operating model

We use a delay difference model to simulate the population dynamics of a single species, and parameterize the model to examine the effects on several species with different characteristics. We assume a linear coastline divided into 100 areas. This model tracks the species biomass in each area each year and accounts for growth of average individuals. Using the Deriso-Schnute biomass model [30], the biomass in patch *i* at the beginning of year *t+1* is 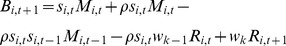
(1)where 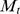 is the biomass in patch *i* after adult movement in year *t*, 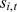 is the annual survival of animals age *k* and older, 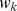 is the average weight of an animal age *k* and older, 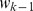 is the average weight of recruits, *k* is the age when the fish can reproduce,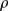 is the Brody growth coefficient that controls the growth rate of individual fish, and 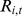 is recruitment in patch *i* in year *t*. The model accounts for two age classes, adults and recruits. All adults for a given species have the same vulnerability to fishing pressure and reproduce. This model assumes that each species is homogeneous across all areas for all biological parameters.

The simulations begin at equilibrium biomass (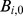), using a specified unfished recruitment in each patch, 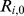 such that
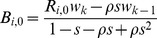
(2)


Annual survival 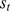 is the product of both natural survival 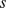 and the survival from fishing mortality. The harvest rate *u* is specified for both pre and post implementation of a no-take zone, for each patch.
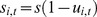
(3)


#### Larval dispersal, recruitment, and adult movement

The number of eggs produced each year is assumed to equal the spawning biomass at the beginning of each year. The larvae are dispersed in a Gaussian fashion, so larvae do drift from one patch to another but the proportion that derive from the source to any given location decreases with distance between the sites. This does not explicitly model larval advection. The relative proportion of fish moving from area *i* to area *j* are defined as
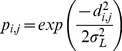
(4)where *d* is the distance between area *i* and area *j*. The model parameter 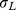 defines the dispersal range for the species. The relative proportions are then normalized so that the total proportion of moving from area *i* to any area sum to one. The settlement of the eggs 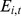 in each patch is then calculated as
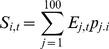
(5)


Adult movement is determined using the same Gaussian movement, with an adult movement parameter of 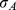.

Density dependent recruitment in each patch is calculated using a Beverton-Holt form [30]

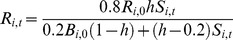
(6)where *h* is the steepness parameter for the species which is describes its productivity level.

### Economic model

#### Fishing

The fishery catch (C) in patch *i*, year *t*, is the product of harvest rate, 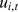, and biomass in each patch and for each year. 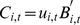
(7)where 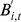 is the biomass of the species after adult movement.

The fishery profit *FV_t_* in year t is given by:
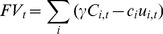
(8)where 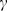 is the price per gram for the species and 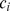 is the cost per unit effort to fish in area *i*.

#### Tourism

**Figure 1:**
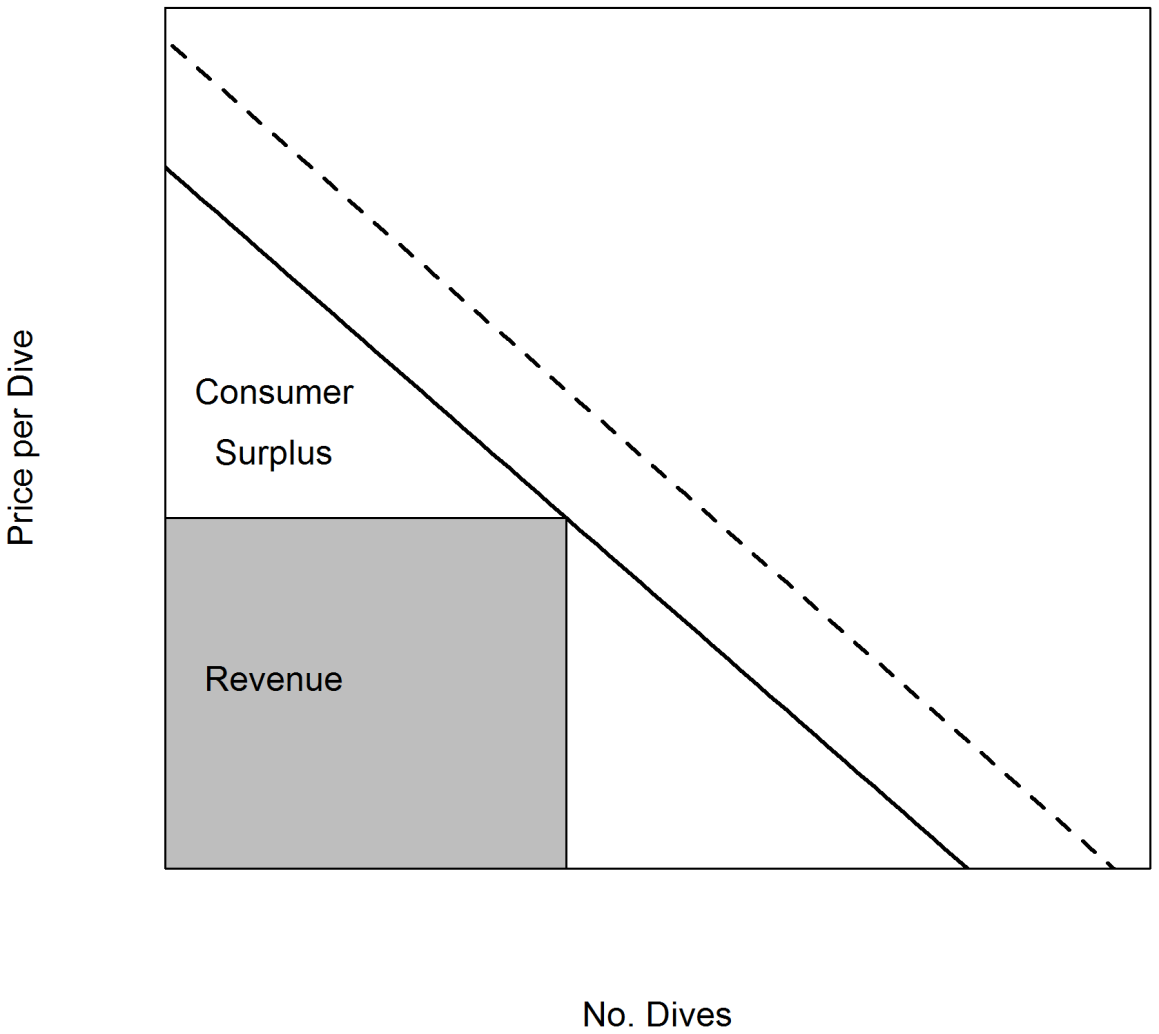
Hypothetical illustration of equation 9 (the solid diagonal line). Revenue and consumer surplus can be calculated as areas under this line and change each year depending on the species biomass and number of dives. The dotted line illustrates equation 9 at higher biomass levels.

Tourism values are often neglected in bioeconomic analyses of marine reserves. We model the marginal value of additional site visits (or “dives”) as follows:
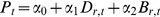
(9)where 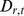 is the number of dives in the reserve and 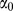, 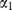, and 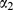 are parameters estimated for each location that the model is applied. Here we focus on economic well-being of divers themselves, and implicitly ignore further ancillary benefits arising from the multiplier effect of tourism revenue in the community. Equation 9 can be used to calculate the number of dive-days demanded for any given price and any given level of fish abundance by solving equation 9 for 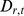. We can also use this to calculate the total value divers place on dives, represented by the consumer surplus (Fig. 1). We expect 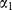
<0, reflecting the fact that additional dives are increasingly less valuable. We expect 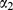
>0, reflecting the fact that a dive's marginal value is positively influenced by additional biomass in the reserve – importantly we assume this effect is linear, which is likely to hold for modest changes in biomass, but may not continue to hold for extremely large increases in biomass. While we focus on the biomass of key species, it is possible that diver demand would also depend on the diversity of fish.

An optimal fee per dive in year *t* (OP_t_) is calculated to maximize the tourism revenue in year *t*. Tourism revenue is defined as the product of the fee per dive and the number of dives in the reserve:



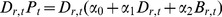
(10).

By taking the derivative of equation 10 with respect to 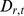 and setting the equation equal to zero, the number of dives that maximize the tourism revenue in year *t* is:



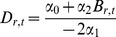
(11).

The optimal fee per dive that maximizes tourism revenue is then calculated by:



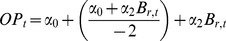
(12).

### Example application

To illustrate the dynamics of the bio-economic model, we present a simulation based on the characteristics from the Medes Islands fishery in Spain. This case study builds from the bio-economic analysis by Merino et al. [31] which focuses on long-run, or equilibrium, effects of reserve implementation. Because one of our main questions concerns the economic returns from reserves, analyzing the inter-annual dynamics is crucial. Our goal is to determine the time period for which the species recovery and economic development of tourism surpass the short term loss in fishing grounds. This case study is provided to illustrate and example of the short term dynamics under this model for a fishery such as the Medes Islands.

The Medes Islands Marine Reserve was created in 1983, and it includes a no-take zone of 51 ha, and partially-protected area of 460 ha where seven local artisanal fishing vessels have exclusive access [31]. We use parameters based on data from this fishery (Table 2) and from a long-term ecological monitoring of the reserve [32] to explore model predictions for this fishery upon implementation of a marine reserve.

**Table 2:** Parameter values for the Medes Islands Marine Reserve example.

Biological Parameters								
Species	*s*	*w_k_*	*w_k-1_*	*ρ*	*R_i,0_*	*σ_L_*	*σ_A_*	*h*
*Mullus surmuletus*	0.66	53.93	0	0.77	52000	2	1	0.75
*Dicentrarchus labrax*	0.9	384.9	0	0.85	6100	2	0.01	0.75

Fishery Parameters								
	*M. surmuletus*		*D. labrax*					
Zone	*u* pre-reserve	*u* post-reserve	*u* pre-reserve	*u* post-reserve				
No-take	0.75	0	0.2	0				
Restricted zone	0.75	0.375	0.2	0.1				
Open access	0.75	0.75	0.2	0.2				

Economic parameters								
**Fishery (Euros)**	**γ (€ kg^−1^)**	**c_i_**						
	9.97	9905						
**Tourism**	**α_0_**	**α_1_**	**α_2_**	**Reserve fee/dive (€)**				
	9.6448	−0.003	.00004	3.5				

Parameter *s* is the annual natural survival rate, *w_k_* is the average weight of an animal age k and older, *w_k-1_* is the average weight of recruits, *ρ* is the Brody growth coefficient, *R_i,0_* is unfished recruitment, *u* is the annual harvest rate, 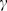 is the price per gram for the species, 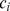 is the cost per unit effort to fish in area *i*, and 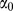, 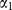, and 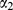 are location specific parameters for the tourism model.

To simulate this reserve system, we use three harvest rates, one for the no-take zone, one for the partial reserve and one for the area with no reserve. The relative size of these areas matches the relative areas of the Medes Islands zones (1%, 12% and 87%). In these areas we simulate the biological dynamics of two representative species and their change in biomass over time. We use the striped red mullet (*Mullus surmuletus*) to represent the species important to the fishery and European seabass (*Dicentrarchus labrax*) to represent the species that divers are interested in seeing in the water.

We conducted a second simulation mirroring management changes that occurred at the Medes Islands Marine reserve. In 1990, the Catalan parliament passed a law that expanded the protection and established tools for more effective conservation management [33]. To prevent the deleterious impact of an excessive number of divers on the fragile benthic communities of the Medes Islands [34], the number of divers was reduced to a maximum of 450 per day. The number of diving centers was regulated, and each diving center has a dive quota; for divers using their own boat there is a first-come first-serve system. These measures were made effective in 1991. Therefore we capped the number of dives in our model in 1991, using a fixed dive fee of € 3.5.

## Results

**Figure 2:**
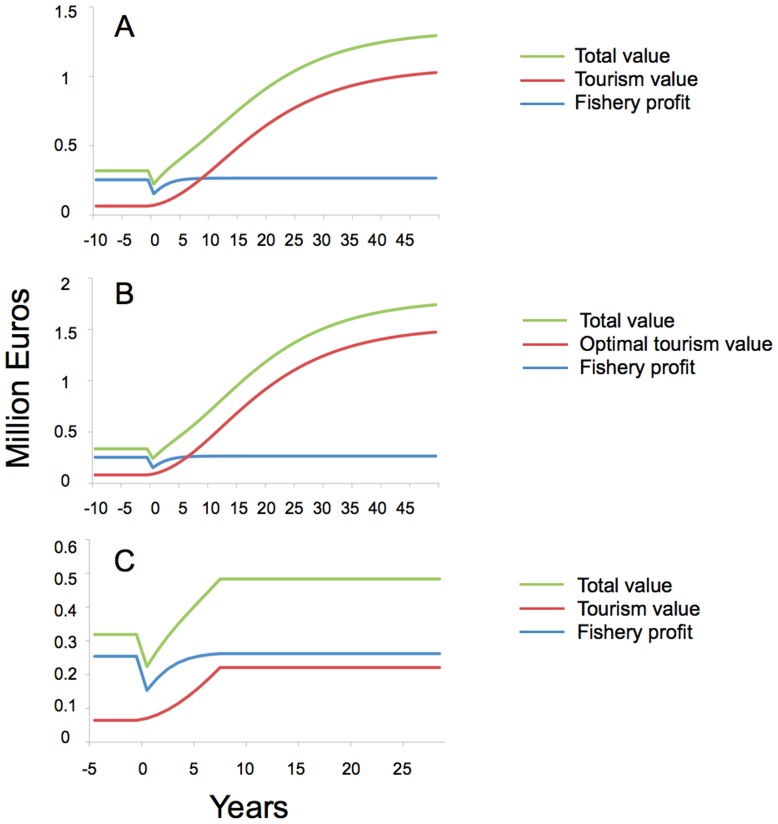
Example simulation based on the Medes Islands marine reserve. A) The reserve is implemented in year zero and the fishery profit and total value (fishery and tourism combined) show short term losses before long term gains. The tourism value increase monotonically over time after implementation of the reserve. B) Medes Islands example with optimal fee per dive calculated each year. C) Medes Islands example capping the number of dives as those in 1991, to simulate actual management changes.

The simulation of the Medes Islands marine reserve was run for 100 years before the implementation of the no-take and partial no-take zones. The simulation is then run for another 100 years to show the long term effects of implementing the reserve system. Results from the simulation show that there is a short term loss in fishery profit accompanied by a steady increase in the tourism value (Fig. 2a). The tourism value accounts for the fee per dive for visitors as well as the consumer surplus, which represents the additional amount that visitors would have been willing to pay for those dives. The fee per dive is assumed to be the current diver access fee of € 3.5 per dive in the Medes Islands for each year of the simulation. In this example, even if one only considers net benefits (consumer surplus plus fishery value), the reserve more than doubles the value of the marine ecosystem, with more value arising from tourism than from fisheries. The total value of the reserve becomes greater than the pre-reserve value within five years of protection.

As discussed above, there are additional values of the reserve that are not captured by this analysis. One obvious source is the multiplier effect of diver expenditures in the local community (hotels, restaurants, car rentals, dive equipment rental, etc.) While we have omitted these additional sources of value, including them would only serve to further increase the benefit of the reserve (see Discussion for actual economic benefits of the Medes Islands Marine Reserve).

We also consider another simulation where the fee per dive is changed each year by calculating the optimal fee each year to maximize tourism revenue using equation 12. This simulation shows the possibility of increasing the tourism value (Fig. 2b). Parameter values used in this example are listed in Table 2. Many of the biological parameters were calculated from other known parameter values for these species [35]. The steepness, *h*, was assumed to be 0.75, which is approximately the modal for steepness values for a range of species [36]. Initial recruitment values for red mullet (representing all fished species) were estimated using carrying capacity values from Merino et al. [31] and then determining the total carrying capacity from these species that represent 5 percent of total catch [31]. Initial recruitment for European seabass (representing species in dive industry) were estimated using relative abundances between the red mullet and European seabass in the no-take zone [32].

Parameters for the fishery were chosen to illustrate a species that is experiencing a fishing pressure beyond its maximum sustainable yield (red mullet) and a reduced rate for nontargeted fish that are caught as bycatch (European seabass). After the implementation of the reserve system, the fishing pressure is assumed to drop to zero in the no-take zone, is reduced in half in the partially-protected area, and remains the same outside of the reserves. Estimates for fishing costs were based on personal communications with local fishers. Prices per kg of fish were based on prices for the red mullet [31]. Estimates for 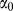, 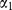, and 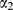
were calculated to reflect the number of divers each year in the marine reserve.

The output of the model was remarkably accurate. When we capped diver numbers at the 1991 level, we obtained 63,000 dives per year, with a revenue generated by diving fees of € 221,000 (Fig. 2c). The actual number of dives conducted in 2009, almost 20 years after the diving quotas had been established, was 67,000 divers, whose diving fees produced a revenue of € 235,500 [33].

## Discussion

An increasing number of studies show that the combined economic benefits of marine reserves (including fishing enhancement, tourism, and ecosystem services) outweigh the costs of creating and maintaining the reserves [31], [37], although to date no reserve has been created with a business plan taking this into account. It is worth noting that while improvements in fisheries may be obtained by other management methods than solely creating a reserve (e.g.; [38]) it is less likely that the tourism benefits would be realized in this way. This is because the tourism benefits with regard to increased fish abundance and size are place-based rather than diffused across all areas where the fish occur. Reserves capitalize on the location specific potential for activities such as diving or other non-extractive uses.

Our bio-economic model shows that fishing revenue increases after the creation of a reserve, and also that tourism revenue surpasses the revenues from fishing. It is worth noting that the total value of the reserve is larger than the pre-reserve value within only five years of protection. This result is in agreement with data on the rapid biological recovery of reserves [39] and short-term local fisheries enhancement [13], [21], [40]. Therefore the typical concern about short-term revenue losses associated to reserve creation, especially for fishers, should be easily addressed with a proper business plan that estimates revenue projections, accounts for costs, and identifies financing mechanisms.

In the Medes Islands Marine Reserve example, before the creation of the reserve, only four diving centers took tourists to the islands, generating a revenue of about € 0.5 million. Presently, the increased abundance of marine life in the reserve supports a thriving tourism industry including diving centers, snorkeling, glass bottom boats, and kayaks. The current diver access fee of € 3.5 per dive (snorkelers, kayakers, and glass bottom boat tourists do not pay access fees) brings in € 234,500 per year, which covers half of the annual budget for the reserve [31], [33]. However, If we add other services (hotels, restaurants) that grew in association with the increase in number of divers, the marine reserve brings a minimum of € 10 million annually to the local economy – and 200 full-time jobs [31], [33]. Before the creation of the reserve there were 21 registered artisanal fishing boats, relative to seven professional boats operating today. The difference in the number of active fishers is due to retirements of ageing fishers, and a shift to more lucrative businesses such as lodging, restaurants, and tourism. Current fishing revenue exceeds € 0.2 million [31]. Although there are no published statistics on the local fisheries economics, interviews with local fishers indicate that revenue before the creation of the reserve was lower than presently. In addition, the areas around the Medes Islands Marine Reserve attract more than 455 recreational fishing boat visits per year, with an average expenditure in fuel, gear and bait of € 800 per boat [41]. Payment for ecosystem services, such as the one afforded by the regeneration of the seagrass *Posidonia oceanica* beds in the reserve could increase income in the reserve; these and other benefits could be added to the model. As our bio-economic model predicts, the aggregate economic value of the Medes Islands Marine Reserve is larger than the costs, and suggests that other reserves in locations with similar tourism opportunities could be designed as revenue and job creators. Our model provides a general framework to estimate costs and benefits and plan consequently.

Uncertainty of future benefits may be one of the larger barriers to reserve formation. The simulation model, paired with local biological and economic data could reduce uncertainty regarding long-run financial benefits of a potential marine reserve. The tourism literature is rich with methodologies to estimate the price-elasticity of demand. The model parameter 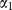 could be estimated in some cases. When decisions must be made quickly and data are lacking, literature estimates from similar locations may still provide useful information on 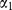. In either case, information on divers price sensitivity paired with the simulation model can give critical information on potential revenues from reserve user-fees. A second critical economic parameter, 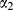, reflects divers preferences for larger and greater numbers of fish. This parameter is not as widely estimated as price-elasticity but it is possible to estimate divers' willingness-to-pay for increases in fish density and size [42].

Other constraints may exist to reserve creation such as capital constraints and the ability of potential beneficiaries to coordinate with those fishers bearing the short-term costs. Essentially, potentially profitable reserves may suffer from incomplete markets. Our example from Medes suggests that even for fisheries alone, the reserve will ultimately have a positive effect. However, in many cases, fishers, the current users might oppose reserve formation even when models and data produce expectations of future profits. The short-term losers may face capital constraints and may have little reason to expect to share in future tourism benefits. Even when future fishery benefits are credible, current fishing interests may not hold secure claims to those future benefits.

There are many potential mechanisms that might resolve capital and coordination constraints. In some cases, improved legal structures guaranteeing current fishers' shares in future benefits may suffice. In other cases, the creation of markets for conservation may be appropriate. Finally, external organizations may wish to speed the formation of reserves by offering a buy-out to reduce fishing effort in the fishing zone, or by financing the loss in fishery value during the time gap between reserve implementation and fishery recovery. Possible financing mechanisms include private investments and public/private partnerships, some of which have proven successful in other social initiatives and businesses. In addition to facilitating the transition from open access to a system with a fraction of the fishing grounds closed as reserves, these mechanisms could cover the costs of creation and management of reserves, making them self-sustaining.

Metrics for success will be critical for long-run benefits to be realized. We cannot conclude that a reserve is failing only because it is not enhancing the catch of one or more species around it (e.g., [43]). That may be simply due to excessive fishing capacity/effort (regardless of the closure of a fraction of the fishing grounds), and the reserve may be too small or located in a sub-optimal location. Furthermore, aggregate benefits afforded by protection may be much greater than the putative loss of fisheries yield. For instance, a fishery targeting a spawning aggregation of large predatory reef fishes will yield lower catches right after the spawning grounds are protected. However, fishing spawning aggregations universally leads to collapse of the aggregations, the populations of the species, and the fisheries they support [44]; whereas the increase in value of the aggregation site through ecotourism and replenishment of adjacent fishing grounds will far offset the short term loss of fishing profit [45]. It is thus essential that, for evaluating the efficacy of a marine reserve, the economic dynamics around the reserve are compared to those in similar areas without reserves.

The economic benefits of marine reserves may be enhanced by additional management around their borders (e.g., TURFs, individual transferable quotas) [46], [47], [48] and co-designing marine reserves with other spatial management measures can further increase benefits [49]. In any case, a business approach could help replicate the success stories in a decentralized way that is not constrained by limited human and financial resources from governments and conservation organizations.
